# Metal Content of Nutritional and Toxic Value in Different Types of Brazilian Propolis

**DOI:** 10.1155/2020/4395496

**Published:** 2020-01-22

**Authors:** Katharine V. S. Hodel, Bruna A. S. Machado, Nathália R. Santos, Renata G. Costa, Jose A. Menezes-Filho, Marcelo A. Umsza-Guez

**Affiliations:** ^1^University Center SENAI CIMATEC, National Service of Industrial Learning, Laboratory of Pharmaceutical's Formulations, Health Institute of Technologies (ITS CIMATEC), Salvador 40110-100, Brazil; ^2^Federal University of Bahia, Laboratory of Toxicology, Pharmacy Faculty, Salvador 40170-290, Brazil; ^3^Federal University of Bahia, Biotechnology, Institute of Health Sciences (ICS), Salvador 40170-290, Brazil

## Abstract

Brazilian raw propolis samples (brown, green, red, and yellow) were investigated to evaluate the content of three elements of nutritional value (Cu, K, and Se) and three toxic metals (As, Cd, and Pb). The propolis samples (*n* = 19) were obtained from different regions of Brazil and analysed by atomic absorption spectrometry after microwave-assisted digestion. A descriptive analysis of the variables was carried out, and nonparametric tests (Kruskal–Wallis or Mann–Whitney) were performed to verify the differences in metal contents. The elemental concentrations of the Brazilian propolis were in the following ranges: As < 0.048–8.47 *μ*g·g^−1^, Pb < 0.006–0.72 *μ*g·g^−1^, Cu 0.57–11.60 *μ*g·g^−1^, Se < 0.041–0.54 *μ*g·g^−1^, and K 0.23–7.94 mg·g^−1^; Cd was below LOD (0.008 *μ*g·g^−1^) in all samples, except one. Seven samples exceeded the limits defined for As or Pb by the Brazilian regulation.

## 1. Introduction

Propolis (bee glue) is a generic name for the resinous substance collected by honey bees (*Apis mellifera*) from various plant sources (substances exuded from wounds in plants, lipophilic materials on leaves and leaf buds, gums, resins, and lattices) and mixing salivary enzymes (*β*-glucosidase) that it is used to seal holes in the honeycombs and smooth out the internal walls [1, 2]. More than 420 different compounds have been characterized so far in propolis, giving it diverse pharmacological properties such as antimicrobial, antioxidative, anticancer, anti-inflammatory, antifungal, antiparasitic, and antiviral activities [2, 3]. Moreover, it is intensively used in the food industry as a supplement and as a folk medicine and by the cosmetic industry [4, 5].

Their color, texture, and chemical composition vary, depending on the location of the hives and local flora [6]. Because of the great diversity of ecosystems and, consequently, floras, besides favourable climate throughout the year, there are different types of propolis in Brazil. Initially, the Brazilian propolis was classified into 12 types based on physicochemical properties (color, texture, and chemical composition) and geographic origin [7]. A 13th type of propolis was reported in the literature in 2007 as Brazilian red propolis, due to its intense red color [8].

The analyses of the inorganic constituents of propolis may be useful for the discrimination and classification of propolis in view of their botanical provenance, type, and level of technological processing [9]; besides, it might be used as a possible tool of biomonitoring with respect to toxic metal contamination [10]. Industrialization and technological advancement have put an increasing burden on the environment by releasing large quantities of hazardous waste, such as toxic metals and organic pollutants [11], generating a growing interest in bioindicator-based techniques for the detection and evaluation of environmental contamination in recent years [12]. Several authors have indicated that bees and their products may be used as biological indicators of the environmental toxic metal pollution [13–16] due to the fact that the honeybees are a good biological indicator as it is widespread and sensitive to environmental changes, monitoring the level of soil, water, plant, and air pollution in areas of several square kilometres [17]. This can bring contaminants to the hive and, consequently, to the manufacture of beekeeping products. Among beekeeping products, propolis can be considered a good biological indicator because, besides being constituted by several organic and inorganic elements according to the geographical area, the sticky nature of gum could be a surrogate of the atmospheric toxic metal contamination [10].

Ferreira et al. [18] analysed the presence of Cu, Fe, K, Mg, Na, Zn, As, Cd, As, Cd, Pb, and Cr metals in ten Brazilian geopropolis samples from the state of Santa Catarina. The results indicated that the presence of different amounts of minerals could attribute a specific geographic location of each geopropolis and also inform the environmental quality of the soil surrounding the beehive. Orsi et al. [19] determined the concentration of toxic metals (Ni, Cr, Hg, Cd, Pb, and Sn) in 106 samples of Brazilian raw propolis, and the transfer rate of these contaminants to ethanolic extracts of propolis was evaluated by atomic absorption spectrophotometry. Despite analysing a large number of samples, the authors of this study did not make clear which types of propolis were analysed. The results showed the presence of all metals analysed in the samples of raw propolis from the states of São Paulo and Minas Gerais.

It is important to note that Brazil is a great producer and exporter of propolis collected by *Apis mellifera* [20]. However, there is little information on the content of trace elements in Brazilian propolis, especially the possible presence of toxic metals by the color types: green, red, yellow, and brown of the Brazilian propolis. Studies about nutrient composition of this bee product are needed to promote its greater production and commercialization in domestic and international markets, through the characterization of the Brazilian propolis quality. In this context, the aim of this study was to evaluate for the first time the content of three elements of nutritional value (Cu, K, and Se) and three toxic metals (As, Cd, and Pb) in four types of propolis (green, red, brown, and yellow) obtained from different regions of Brazil.

## 2. Materials and Methods

### 2.1. Propolis Samples

This study was carried out with 19 representative samples of propolis from regional beekeepers (Table 1). The samples represent four types of Brazilian propolis: brown, green, red, and yellow. The different samples were kindly donated by the companies Apis Jordans (Vitória da Conquista, Bahia, Brazil), Apis Nativa Produtos Naturais (Prodapys, Santa Catarina, Brazil), and Bee Product Natural (Alagoas, Brazil). The group of brown samples was the largest in number of samples, 10 in total, followed by the red group, with 4 samples, as well as the green group, with 4 representatives, and the yellow group with only one sample. Moreover, propolis was obtained from different regions of Brazil: northeast (semiarid region characterized by caatinga biome), central-west, southeast, and southern, and from several states, as shown in the Table 1. In addition, the botanical origin of each crude propolis sample was investigated according to literature data, and these references are also evidenced in Table 1. It is important to note that the samples were collected in different years and belonging to different batches. The samples were stored in a freezer at −10°C until processing.

### 2.2. Sample Preparation

All samples were weighed on an analytical balance (Sartorius CP2245, Gaithersburg, USA) directly into the 25 mL Teflon flask. Then, the samples were mineralized by microwave-assisted digestion (Mars6; CEM, Matthews, USA). Initially, 4 mL of concentrated ultrapure nitric acid (HNO_3_; Merck, Darmstadt, Germany) were carefully added to 200 mg of mass of each sample. After 10 minutes at room temperature, 1 mL 30% v.v^−1^ of hydrogen peroxide (H_2_O_2_; Synth, São Paulo, Brazil) was added before subsequent digestion in the microwave oven. The digestion program chosen was the built-in method: power 1030–1800 W; total duration time including cooling 40 minutes; and maximum temperature 200°C. The propolis samples were placed in this category because of its texture characteristics. Afterwards, the mineralized samples were transferred into graduated centrifuge tubes and volume adjusted to 10 mL with ultrapure water (Milli Q, Millipore, Bedford, USA).

### 2.3. Quantitative Determination

Reference materials, reagent blanks, and propolis mineralized samples were analysed by Graphite Furnace Atomic Absorption Spectrometry (GFAAS) with Zeeman background correction (Spectra AA 240Z and GTA-120 Varian, Mulgrave, Australia) to determine Cd, Pb, Se, and As and Flame Atomic Absorption Spectrometry (FAAS) (Varian Model 55B, Palo Alto, USA) to determine Cu and K. The detection of metals in both equipment occurred according to the analytical parameters and temperature program indicated in the equipment manual for each metal analysed. The program temperature for each metal analysed by GFAAS is detailed in Table S1. Each sample was analysed in duplicate, and the spectrometer performed two readings of each sample, calibrator, and reference material. The average metal levels were measured in the reagent blank and subtracted from the metal content measured in the samples and reference material [25]. The resulting concentrations of each metal in the 19 propolis samples were expressed in micrograms (As, Cd, Pb, and Se) or milligrams (Cu and K) of metal per gram (dry weight), and then, the average metal concentration per sample was calculated.

### 2.4. Analytical Quality Assurance

For quality control purposes, certified reference materials were used for validation of the analytical procedures because it plays an important role in terms of accuracy and reliability. The Standard Reference Materials (SRM) used were NIST 1570a Spinach Leaf and NIST 1566b Oyster Tissue. These materials were obtained from National Institute of Standards and Technology (NIST, Gaithersburg, USA). The NIST Spinach Leaf was used for K and Cu analyses, whereas NIST Oyster Tissue was used for Cd, As, Se, and Pb analyses. In order to confirm method's performance, the analytical parameters were calculated as the limits of detection (LOD) and quantification (LOQ), where the LOD was established as the blank's average (*n* = 8) plus three times the standard deviation (SD) and the LOQ was the blank's mean plus ten times the SD [26], precision (coefficient of variation <10%), and accuracy (85–115%). The values of precision and accuracy were compared with values of guideline from the European Medicine Agency [27] and the Brazilian National Sanitary Surveillance Agency [28].

### 2.5. Data Analysis

Metal concentrations (As, Cd, Pb, Se, Cu, and K) in 19 propolis samples were evaluated by the chemometric tool principal components analysis (PCA) in order to obtain the correlation between the propolis samples according to their levels of toxic metals. PCA analysis was carried out by PAST software version 3.26 (Oslo, Norway). Additionally, a descriptive analysis of the variables was carried out. The values of median, standard deviation, minimal, and maximum for each metal were calculated according to the type of propolis: brown, green, and red. As the yellow type had only one representative, the values of the descriptive analyses were considered constant. Nonparametric tests of Kruskal–Wallis and Mann–Whitney were performed to verify if the distribution and the medians of the metals are the same between the types of propolis, respectively. The statistical analyses were calculated using the software IBM SPSS Statistics for Windows (Chicago, Illinois, USA).

## 3. Results and Discussion

For this study, six metals (As, Cd, Cu, K, Pb, and Se) were evaluated. The microwave-assisted digestion technique was used in this work to provide better mineralization since it reduces sample preparation time and the problems associated with loss of more volatile components and contamination.

The quality assurance data (Table 2) were obtained in the calculations of limits of detection and quantification, precision, and accuracy for each metal analysed. We concluded that the results are satisfactory because the values observed are within those recommended by the two agencies, where the precision should not exceed 15% and that accuracy percentages should be close to 100%.

In a general way, the raw propolis samples had different ranges in the mineral composition (Table 3). For being constituted basically by a resinous hive substance containing beeswax, plant exudates, and salivary secretions from bees, the propolis can be contaminated by metals by different sources such as bees, air, water, plants, and soil [26–28]. Propolis contamination by soil and plants are closely related since soil-plant transfer of metals is controlled by numerous factors related to plant physiology, such as plant type, rate and type of root secretions, root surface area and transpiration, and soil properties, such as texture, pH, and cation exchange capacity [9, 29]. The presence of metals in bees is associated with the direct deposit of toxic metals present in the atmosphere on the hairy body of the bees or can reach the insect by the nectar, the pollen, the honeydew, or through the water during foraging [17].

It is important to note that the origin of contamination of metals is associated mainly with anthropogenic factors such as industrial activities, mining, increased urbanization, fertilizer, and pesticides use but also may be related to natural factors such as erosion and leaching from geological formations [9, 28, 30]. Some elements play important roles in animal and plant organisms, such as Se, Cu, and K, acting on the regulation of metabolic pathways and physiological processes [31]. The toxicity of these elements is associated with concentration in living organisms [32], while others are considered inorganic contaminants, such as As, Cd, and Pb, and its presence in certain concentrations in organisms can have adverse effects [33].

Arsenic was one of the toxic metals determined. Fourteen samples out of nineteen presented As levels below the procedural limits of detection (0.048 *μ*g·g^−1^). In samples that As was detected, the values varied between 0.05 and 8.47 *μ*g·g^−1^. Samples P10 and P13 presented the highest levels of this metal, with levels of 8.47 *μ*g·g^−1^ and 4.79 *μ*g·g^−1^, respectively. Both samples are of brown type and came from the Southern Santa Catarina State. Zoffoli et al. [34]analysed the presence of heavy metals in tobacco fields in Brazil's Southern Region, including Santa Catarina state, and showed that the use of fertilizers is a major source of arsenic, as well as other metals not essential for plants. Thus, possible agricultural activities in the collection areas of propolis or in regions within the bee flying spectrum may be associated with the presence of As in the samples. Matin et al. [10] analysed the presence of toxic metals, among them As, in 5 samples of propolis from the industrial district of Izmir, Turkey. The arsenic levels in the samples ranged from 0.019 to 0.578 *μ*g·g^−1^ levels lower than those observed in the present work.

Only the sample P10 had the detectable cadmium level, about 0.03 *μ*g·g^−1^. One of the sources of propolis contamination is the plants in which bees collect the resins. Divan et al. [35] showed that *Baccharis dracunculifolia*, one of the sources of resin collection for the production of brown propolis [23], has a moderate capacity to accumulate Cd, transforming this plant into a possible source of contamination. The presence of Cd in this sample may be related to the use of agricultural supplies, such as fungicides, in plantations in Santa Catarina state [34]. Sattler et al. [31] analysed the presence of several toxic metals, among them is Cd, in different pollen samples (apicultural products originated from different botanical sources, such as propolis) from the state of Rio Grande do Sul, in Brazil. The variation found in pollen samples with respect to Cd was lower than that found in sample P10.

As expected, copper was found in all samples, and the concentration varied from 0.57 to 11.60 mg·g^−1^. The samples P09 and P10, of the brown group, presented the highest levels of this microelement with 7.76 and 11.60 mg·g^−1^, respectively, and the sample P19, of the yellow group, presented the lowest level. The presence of Cu in all samples can be justified by the indispensable role of this metal in all organisms, and in this way, the presence of Cu can be detected in possible sources of contamination of propolis, such as plants and bees, since it plays key roles in several biochemical and physiological processes. Furthermore, Cu is important for propolis because it may be assumed that phenolic compounds present in propolis [36] tend to chelate metals such as Cu, catalysing components of chemical reactions which originate free radicals or chelates toxic metals, forming complexes that cause color development [37]. On the contrary, the presence of copper in the soil, besides natural concentration that depends on its concentration in rocks, can be associated with the use of fertilizers and fungicides in agricultural activities [38]. In our study, the samples of Southern Brazil presented the highest levels of Cu. This could be associated with the high concentration of this metal in the soil due to anthropogenic activities, such as mining [39, 40]. Dogan et al. [41] evaluated the presence of Cu, in addition to other elements, in 7 propolis samples from different geographic regions of Turkey. All the samples indicated Cu in their composition, and the levels found in the samples were lower when compared to the ones in this study, with values ranging from 0.0045 to 0.0096 mg·g^−1^.

Eleven samples had the presence of selenium detected, with the concentration ranging from 0.19 to 0.54 *μ*g·g^−1^, while in six of them, Se levels were below the procedural LOD set at 0.041 *μ*g·g^−1^. Bonvehí and Bermejo [37] considered the Se values found in the 25 samples of raw propolis from Spain limited (<0.112 *μ*g·g^−1^). The values found by Golubkina et al. [42] in samples of propolis from regions of Moldavia, Moscow, and Mongolia ranged from 0.6 to 2.18 *μ*g·g^−1^. This variation of results can be attributed to the different constitutions of Se in soils of different parts of the world [43], and the presence of Se in Brazilian soil may be related to the use of fertilizers [44].

Potassium was the element that showed presence in all the samples, with levels ranging from 0.23 to 7.94 mg·g^−1^. The concentration of K in the samples can be justified by the presence of this alkaline metal in abundance in the bees [45] and plants [46], possible sources of minerals of propolis. Finger et al. [16] analysed the presence of K in forty-two raw propolis samples from State of Paraná, Brazil. The values found were similar to our results, which ranged from 2.63 to 11.35 mg·g^−1^. However, the values found by Korn et al. [47] in samples of propolis from several regions of Bahia were smaller, with values between 0,199 and 1,892 mg·g^−1^.

**Figure 1:**
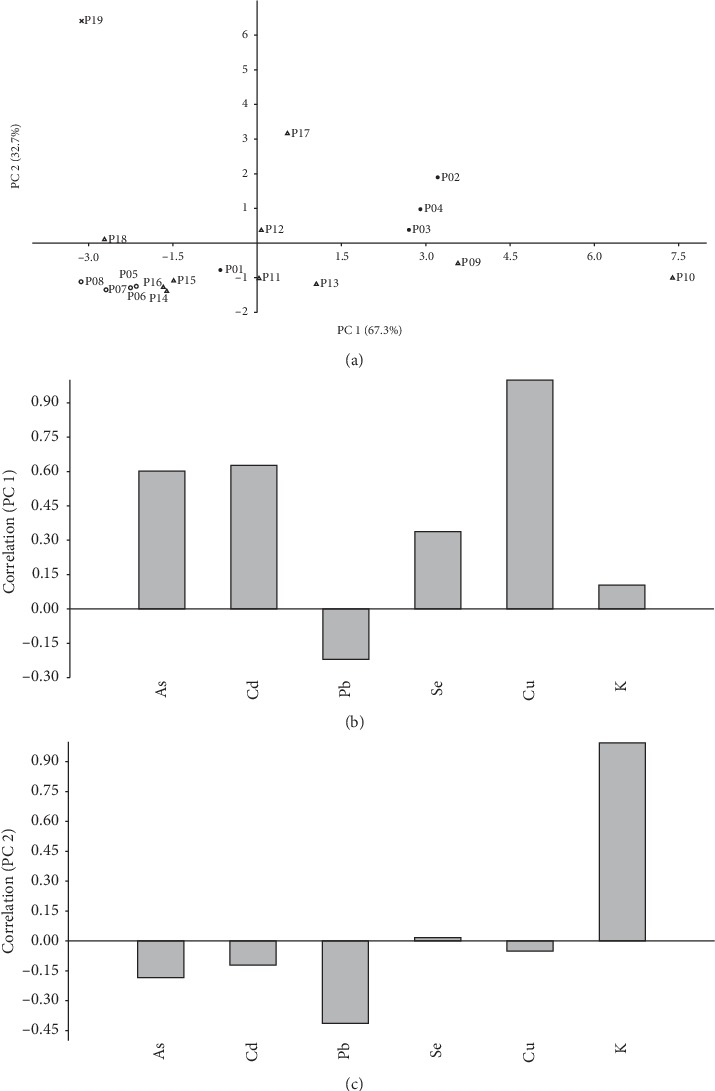
Principal component analysis for 19 propolis samples: (a) graph of scores; (b) graph of loadings for principal component 1; (c) graph of loadings for principal component 2.

Figure 1(a) shows the correlation between PC 1 and PC 2, the main components of the propolis studied in this work. PC 1 varied about 67.3%, and PC 2 varied 32.7% of the total variance of data. PC 1 was positively correlated with As, Cd, Se, K, and Cu and negatively correlated with Pb (Figure 1(b)). Cu presented the highest positive correlation. Regarding PC 2, it presented a higher positive correlation with K and negative correlation with As, Cd, Pb, and Cu (Figure 1(c)). Sample P19 presented the highest score in the positive axis of PC 2 and represents an extreme due to the high concentration of K. Sample P10 presented a high concentration in Cu and represents an extreme in the positive axis of PC 1. These data highlights the strong influence that these elements has in the mineral composition of the propolis evaluated, which is related to its physiologic importance for bees and plants, as well as their presence in the environment for natural or anthropogenic reasons. The results of the statistical analysis are shown in Table 4. As only one propolis sample had a detectable level of Cd, data for this element are not included. The nonparametric Mann–Whitney test showed that the medians of each metal were the same between each type. The Kruskal–Wallis test showed that, only for As, Pb, and Se, the distribution is the same between the categories of each type, whereas for Cu and K, this hypothesis was rejected.

Brazilian propolis is highly valued in the international market, and Brazil is one of the largest exporters of propolis in the world [48]. This economic importance meant that, in 2001, Ministry of Agriculture, Supply and Livestock (*Ministério da Agricultura*, *Pecuária e Abastecimento* (MAPA)) established a technical protocol to define the identity of bee products and minimal parameters for their quality control [49]. The maximum allowable levels for metals in raw propolis is not set in this normative, but it states that inorganic contaminants, such as Cd, As, and Pb, must not be present in propolis in higher quantities than those defined by the regulation RDC number 42 by ANVISA for honey [14, 50]. The limits for Cd is 0.1 mg·kg^−1^ (0.1 *μ*g·g^−1^) and 0.3 mg·kg^−1^ (0.3 *μ*g·g^−1^) for Pb and As. Based on these guidelines, it is not recommended that the samples P09 and P13 be consumed, as they exceeded the limits for As, and samples P07, P12, P14, P16, and P18 exceeded the limits for Pb. As far as propolis type is concerned, the brown propolis had the arsenic mean level (1.36 *μ*g·g^−1^) approximately four times higher than the Brazilian guideline and brown and red propolis reaching borderline values accepted for lead.

Although raw propolis is little consumed in Brazil, it is important to evaluate whether the ingestion of these samples does not pose a human health risk. Thus, it was calculated the provisional tolerable weekly intake (PTWI), based on the report by the Joint FAO/WHO Expert Committee on Food Additives [51], for the samples P08 and P10 had the highest levels of Pb and As, respectively. To calculate the PTWI parameter, we considered the consumption of 1 g of raw propolis/day, body weight 60 kg for adults, and the average values of each inorganic contaminant found in the respective samples. The calculated safe values for Pb and As intake should be less than 25 and 15 *μ*g·kg^−1^ body weight, respectively. The results obtained showed that average consumption of As and Pb due to the ingestion of the samples P08 and P10 was lower than the tolerable levels for the PTWI parameter (Table 5).

## 4. Conclusions

The evaluation of toxic metals in propolis by GFAAS and FAAS contributed to elucidate the mineral content of the 19 raw propolis samples, which were obtained from several regions of Brazil. The results showed that detectable levels of Cu and K were observed in all samples and As, Cd, Pb, and Se in 26.3%, 5.2%, 73.9%, and 57.9% of the samples, respectively. These results suggest that raw propolis samples can be used as bioindicators of environmental pollution by metals since they can be modified by the concentration of trace elements in beehives and their surroundings. Seven samples exceeded the limits defined for As or Pb by Brazilian regulation for honey since there is no specific regulation for propolis although, according to the parameter PTWI, the consumption of Pb and As through the ingestion of samples P08 and P10 did not offer possible adverse health effects. It is important to emphasize that this is the first investigation of metals in trace levels in four different types of propolis and from several regions in Brazil, providing a valuable contribution to the establishment of a Brazilian technical regulation for propolis that may be consumed and exported with safety guarantee worldwide.

## Figures and Tables

**Table 1:** Characteristics of the 19 raw propolis samples evaluated.

Sample code	Origins (state/region)	Botanical sources	References	Type	Harvest year	Weight (mg)
P01	Bahia (northeast)	*Baccharis dracunculifolia*	Pedrazzi et al. [21]	Green	2015	205.4
P02	Minas Gerais (southeast)	*Baccharis dracunculifolia*	Pedrazzi et al. [21]	Green	2014	233.5
P03	Paraná (southern)	*Baccharis dracunculifolia*	Pedrazzi et al. [21]	Green	2014	230.6
P04	Minas Gerais (southeast)	*Baccharis dracunculifolia*	Pedrazzi et al. [21]	Green	2014	204.4
P05	Sergipe (northeast)	*Dalbergia ecastaphyllum*	Piccinelli et al. [22]	Red	2014	202.8
P06	Alagoas (northeast)	*Dalbergia ecastaphyllum*	Piccinelli et al. [22]	Red	2014	221.2
P07	Bahia (northeast)	*Dalbergia ecastaphyllum*	Piccinelli et al. [22]	Red	2015	214.7
P08	Alagoas (northeast)	*Dalbergia ecastaphyllum*	Piccinelli et al. [22]	Red	2016	208.8
P09	Santa Catarina (southern)	*Baccharis dracunculifolia* and *Vernonia polyanthes*	Heimbach et al. [23]	Brown	2012	213.9
P10	Santa Catarina (southern)	*Baccharis dracunculifolia* and *Vernonia polyanthes*	Heimbach et al. [23]	Brown	2014	240.1
P11	Bahia (northeast)	*Hyptis divaricata*	Park et al. [7]	Brown	2015	205.4
P12	Santa Catarina (southern)	*Baccharis dracunculifolia* and *Vernonia polyanthes*	Heimbach et al. [23] and Park et al. [7]	Brown	2014	211.9
P13	Santa Catarina (southern)	*Baccharis dracunculifolia* and *Vernonia polyanthes*	Heimbach et al. [23] and Park et al. [7]	Brown	2013	231.2
P14	Santa Catarina (southern)	*Baccharis dracunculifolia* and *Vernonia polyanthes*	Heimbach et al. [23] and Park et al. [7]	Brown	2014	211.7
P15	Santa Catarina (southern)	*Baccharis dracunculifolia* and *Vernonia polyanthes*	Heimbach et al. [23] and Park et al. [7]	Brown	2014	231.2
P16	Rio Grande do Sul (southern)	*Baccharis dracunculifolia* and *Vernonia polyanthes*	Heimbach et al. [23] and Park et al. [7]	Brown	2014	205.4
P17	Santa Catarina (southern)	*Baccharis dracunculifolia* and *Vernonia polyanthes*	Heimbach et al. [23] and Park et al. [7]	Brown	2014	210.5
P18	Paraná (southern)	*Baccharis dracunculifolia* and *Vernonia polyanthes*	Heimbach et al. [23] and Park et al. [7]	Brown	2013	227.7
P19	Mato Grosso do Sul (central-west)	Unknown	Salatino and Salatino [24]	Yellow	2016	212.8

**Table 2:** Performance characteristics of the analytical method.

Metal	Parameters			
	LOD	LOQ	Accuracy (%)	Precision (%)
As	0.048 *μ*g·g^−1^	0.145 *μ*g·g^−1^	88.7	4.24
Cd	0.008 *μ*g·g^−1^	0.022 *μ*g·g^−1^	93.6	3.05
Pb	0.006 *μ*g·g^−1^	0.017 *μ*g·g^−1^	99.8	9.42
Se	0.041 *μ*g·g^−1^	0.119 *μ*g·g^−1^	88.1	1.95
Cu	0.016 mg·g^−1^	0.049 mg·g^−1^	94.3	0.89
K	0.011 mg·g^−1^	0.031 mg·g^−1^	102.6	5.78

**Table 3:** Average concentrations of metals in the 19 raw propolis samples analysed.

Sample code	As (*μ*g·g^−1^)	Cd (*μ*g·g^−1^)	Pb (*μ*g·g^−1^)	Se (*μ*g·g^−1^)	Cu (mg·g^−1^)	K (mg·g^−1^)
P01	<0.048	<0.008	0.11	0.30	3.58	0.95
P02	<0.048	<0.008	<0.006	<0.041	7.22	3.90
P03	0.07	<0.008	<0.006	0.43	6.82	2.35
P04	<0.048	<0.008	<0.006	0.54	6.98	2.96
P05	0.30	<0.008	0.03	<0.041	2.10	0.37
P06	<0.048	<0.008	0.13	<0.041	2.00	0.32
P07	<0.048	<0.008	0.38	<0.041	1.57	0.23
P08	0.05	<0.008	0.72	<0.041	1.11	0.43
P09	<0.048	<0.008	0.27	<0.041	7.76	1.46
P10	8.47	0.030	0.25	<0.041	11.60	1.32
P11	<0.048	<0.008	0.07	0.39	4.26	0.77
P12	<0.048	<0.008	0.66	0.31	4.20	2.16
P13	4.79	<0.008	0.20	0.29	5.29	0.68
P14	<0.048	<0.008	0.55	0.34	2.65	0.28
P15	<0.048	<0.008	0.11	0.24	2.75	0.59
P16	<0.048	<0.008	0.63	0.19	2.58	0.39
P17	<0.048	<0.008	<0.006	0.36	4.46	4.98
P18	<0.048	<0.008	0.04	0.29	1.44	1.63
P19	<0.048	<0.008	<0.006	<0.041	0.57	7.94

**Table 4:** Statistics of metal contents in the raw propolis samples according to color types (*n* = 18). The K–W test was estimated at a significance level of 0.05.

Statistics	Propolis color	*n*	As (*μ*g·g^−1^)	Pb (*μ*g·g^−1^)	Se (*μ*g·g^−1^)	Cu (mg·g^−1^)	K (mg·g^−1^)
Median	Brown	10	<0.048	0.23	0.29	4.23	1.04
	Green	4	<0.048	0.01	0.36	6.90	2.65
	Red	4	<0.048	0.25	<0.04	1.78	0.34

Minimum	Brown		<0.048	<0.006	<0.041	1.44	0.28
	Green		<0.048	<0.006	<0.041	3.58	0.95
	Red		<0.048	0.03	<0.041	1.11	0.23

Maximum	Brown		8.47	0.66	0.39	11.60	4.98
	Green		0.07	0.11	0.54	7.22	3.90
	Red		0.3	0.72	0.01	2.10	0.43

**Table 5:** Estimated value of provisional tolerable weekly intake (PTWI), considering the daily consumption of 1 g of raw propolis and an adult body weight of 60 kg.

Metal	Sample	PTWI (*μ*g·kg^−1^ body weight)	FAO/WHO (*μ*g·kg^−1^ body weight)
As	P10	0.99	15
Pb	P08	0.08	25

## Data Availability

The data used to support the findings of this study are included within the articlea.

## Supplementary Materials

Supplementary Materials Table S1: spectrometric and temperature program of each metal analysed by GFAAS.
