# Supplementary material for: Metal Content of Nutritional and Toxic Value in Different Types of Brazilian Propolis
Source: ScientificWorldJournal. 2020 Jan 22;2020:4395496. doi: 10.1155/2020/4395496 (PMC7204097; doi:10.1155/2020/4395496)
Supplement: Supplementary Materials — Table S1: spectrometric and temperature program of each metal analysed by GFAAS. [file 4395496.f1.docx]

## Supplementary Materials

Table S1. Spectrometric and temperature program of each metal analyzed by GFAAS.

|  |  | As | Pb | Cd | Se |
| --- | --- | --- | --- | --- | --- |
| Temperature (ºC) | Dry | 95 | 120 | 120 | 120 |
|  | Ash | 120 | 750 | 250 | 1000 |
|  | Atomization | 1400 | 2400 | 1800 | 2600 |
| Wavelength (nm) |  | 193.7 | 283.2 | 228.8 | 196.0 |
| Slit width (nm) |  | 0.5 | 0.5 | 0.5 | 1.0 |
